# Rapid formation of new peripheral arteriovenous malformations post-interventions

**DOI:** 10.11604/pamj.2019.33.128.19258

**Published:** 2019-06-21

**Authors:** Gaurav Gheewala, Iqbal Ratnani

**Affiliations:** 1 Department of Anesthesiology and Critical Care, DeBakey Heart and Vascular Center, Houston Methodist Hospital, Houston, TX, USA

**Keywords:** AVM, arteriovenous malformation, peripheral arteriovenous malformation

## Image in medicine

A 17-year-old male with a history of recurrent peripheral arteriovenous malformations (PAVMs) of right chest, axilla, and right upper extremity (RUE), presented after having multiple procedures for PAVMs and complex right shoulder wound. Being managed conservatively at first, he later underwent diagnostic angiogram and biopsy which shows ectatic vessels, hence, onyx embolization of right subclavian, axillary, and brachial arteries was performed (A). Within two years, due to his necrotic right shoulder wound with osteomyelitis of humeral head, he underwent RUE amputation and resection of newly developed right chest PAVMs with further onyx embolization post angiogram (B). He also required series of reconstructive procedures including tissue expander placement and subsequent muscle flap to necrotic anterior right chest wound which was measured 14x3cm. Patient was subsequently discharged from the hospital after six months and is being followed by vascular and plastic surgery team. Other than cerebral AVMs, PAVMs are rare and get diagnosed by history, angiography, imaging studies, or biopsy. Treatment options are embolization, and/or resection, however, these interventions lead to formation of new AVMs from increasing flow to the surrounding vasculature. Therefore, patients with AVMs remain at high risk for complications despite appropriate interventions.

**Figure 1:**
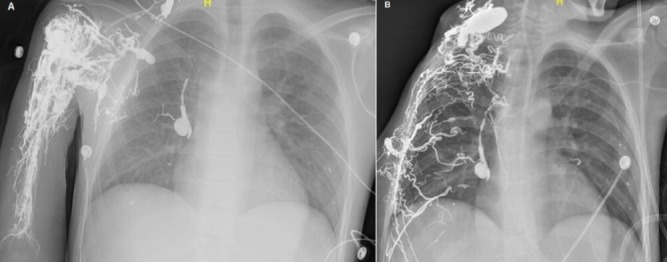
A) onyx embolization of right subclavian, axillary, and brachial arteries; B) right upper extremity amputation with further onyx embolization

